# Increased intracellular activity of MP1102 and NZ2114 against *Staphylococcus aureus in vitro* and *in vivo*

**DOI:** 10.1038/s41598-018-22245-5

**Published:** 2018-03-09

**Authors:** Xiao Wang, Xiumin Wang, Da Teng, Ruoyu Mao, Ya Hao, Na Yang, Zhanzhan Li, Jianhua Wang

**Affiliations:** 1 Key Laboratory of Feed Biotechnology, Ministry of Agriculture, Beijing, 100081 P. R. China; 2 Gene Engineering Laboratory, Feed Research Institute, Chinese Academy of Agricultural Sciences, Beijing, 100081 P. R. China

## Abstract

Treatment of *Staphylococcus aureus* infections remains very difficult due to its capacity to survive intracellularly and its multidrug resistance. In this study, the extracellular/intracellular activities of plectasin derivatives-MP1102/NZ2114 were investigated against three methicillin-susceptible/-resistant *S. aureus* (MSSA/MRSA) strains in RAW 264.7 macrophages and mice to overcome poor intracellular activity. Antibacterial activities decreased 4–16-fold under a mimic phagolysosomal environment. MP1102/NZ2114 were internalized into the cells via clathrin-mediated endocytosis and macropinocytosis and distributed in the cytoplasm; they regulated tumor necrosis factor-α, interleukin-1β and interleukin-10 levels. The extracellular maximal relative efficacy (E_max_) values of MP1102/NZ2114 towards the three *S. aureus* strains were >5-log decrease in colony forming units (CFU). In the methicillin-resistant and virulent strains, MP1102/NZ2114 exhibited intracellular bacteriostatic efficacy with an E_max_ of 0.42–1.07-log CFU reduction. In the MSSA ATCC25923 mouse peritonitis model, 5 mg/kg MP1102/NZ2114 significantly reduced the bacterial load at 24 h, which was superior to vancomycin. In MRSA ATCC43300, their activity was similar to that of vancomycin. The high virulent CVCC546 strain displayed a relatively lower efficiency, with log CFU decreases of 2.88–2.91 (total), 3.41–3.50 (extracellular) and 2.11–2.51 (intracellular) compared with vancomycin (3.70). This suggests that MP1102/NZ2114 can be used as candidates for treating intracellular *S. aureus*.

## Introduction

*Staphylococcus aureus* (*S. aureus*), which is present in both healthy and diseased humans and animals, is a commensal opportunistic pathogen^1^. In human beings, *S. aureus* is a major pathogen that causes significant morbidity and mortality in both community- and hospital-acquired infections^2^. Meanwhile, as a leading cause of infections in some economically important livestock species, *S. aureus* infection has also become an economic burden for the livestock industry^3^. Hitherto, *S. aureus* infection remains very difficult to treat due to multidrug resistance and its intracellular accumulation in host cells^4,5^. *S. aureus* is termed a facultative intracellular pathogen based on accumulating evidence of its ability to survive within the host’s cells. Intracellular *S. aureus* has immune-evasive strategies to escape the detection of professional phagocytes^5^, leading to a lethal metastatic infection. Chronic and recurrent infections may be related to the maintenance of an intracellular pool of bacteria^6^. Furthermore, intracellular bacteria may be protected from high concentrations of extracellular antibiotics, which in turn enhance the risk of developing drug resistance^7^.

Antibiotics are primary drugs that can prevent bacterial infection and significantly contribute to human and animal health. Many studies have been performed regarding the activities of antibiotics such as vancomycin and linezolid against different intracellular *S. aureus* strains in various cellular models^8–10^. However, the poor intracellular bactericidal activity of antibiotics such as oxacillin, levofloxacin, garenoxacin, moxifloxacin and oritavancin towards intracellular bacteria is affected by the following problems: i) low levels of cellular accumulation (linezolid, β-lactams, and gentamicin), which is only partially and non-consistently predictive of activity; ii) acidic environments (aminoglycosides) and iii) binding to intralysosomal constituents (oritavancin)^11^. Therefore, some antibiotics have to be used at large extracellular concentrations to achieve significant activity, which may in turn increase the problem of drug resistance and residual and side effects. Thus, a series of problems have promoted the need for advanced and/or alternative antimicrobial drugs.

Antimicrobial peptides (AMPs) are widely distributed host defense molecules^12^ and defensin-like peptides are a major AMP family. Plectasin from *Pseudoplectania nigrella* is a recently reported novel defensin-like peptide that has potent antimicrobial activities against *S. aureus*, including some antibiotic-resistant strains such as methicillin-resistant *S. aureus* (MRSA)^13^. It has been demonstrated that the intracellular antibacterial activity of plectasin was maintained even though its efficacy was inferior to that of extracellular killing^14^. The novel plectasin variant NZ2114 displayed significantly more potent activities than its parental peptide^15,16^. Moreover, NZ2114 had extracellular and intracellular activities, which was more effective than vancomycin against the intracellular forms of susceptible bacteria, though the intracellular activity was weaker than the extracellular activity^17^. However, the cellular accumulation and internalization mechanisms of plectasin and NZ2114 still remain unclear. Additionally, to further improve the antibacterial activities and properties of NZ2114, a new derivative, MP1102 (N9E, L13V, and R14K), was designed in our previous study. Compared to NZ2114, MP1102 had stronger activity against *S. aureus* and resistance to pepsin, indicating potential as a new antimicrobial agent^18,19^. However, it still remains unclear whether MP1102 has intracellular activity toward *S. aureus*.

In this study, the internalization, distribution and mechanism of MP1102 and NZ2114 uptake into RAW 264.7 macrophages were characterized. The potential antibacterial activities of the two peptides were further investigated against three intracellular *S. aureus* strains (including methicillin-susceptible *S. aureus* (MSSA) ATCC25923, MRSA ATCC43300 and clinical high virulent CVCC546)^20–22^ in broth and in RAW 264.7 macrophages (Supplementary Table 2). Furthermore, the release of cytokines regulated by MP1102 and NZ2114 was assessed in *S. aureus*-infected RAW 264.7 macrophages and their intracellular therapeutic efficacy was determined in an *in vivo* mouse peritonitis model.

## Results

### *S. aureus* was phagocytosed by RAW 264.7 macrophages

**Figure 1:**
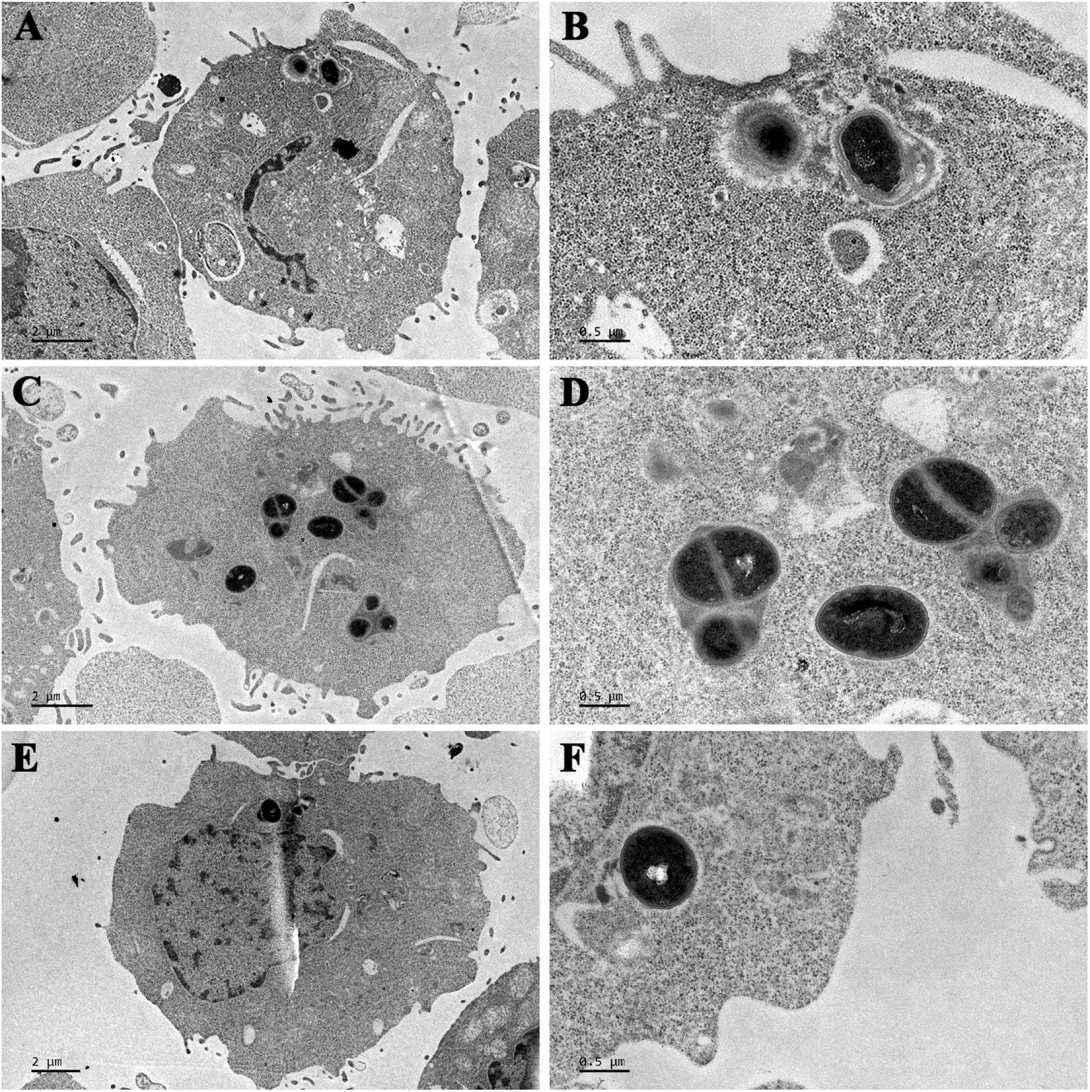
Morphologies of *S. aureus* in RAW 264.7 cells. The cells were challenged with MSSA ATCC25923 (**A,B**), MRSA ATCC43300 (**C,D**) and virulent *S. aureus* CVCC546 (**E,F**) at a multiplicity of infection (MOI) of 100:1 (bacteria to macrophages) and observed by TEM after 0.5 h of phagocytosis. (**B**,**D** and **F**) are enlarged (**A**,**C**, and **E)** respectively.

To investigate the localization of intracellular *S. aureus*, transmission electron microscopy (TEM) was used to analyze RAW 264.7 macrophages infected with MSSA ATCC25923, MRSA ATCC43300 and high virulent multidrug resistant CVCC546 (*spa* type t034) (Supplementary Table 2 and 3, Figure S1). The results showed that the three pathogens could enter RAW 264.7 macrophages without damaging the host cells and that the intracellular bacteria were located in small vacuoles (tight phagosomes) (Fig. 1A and B) and the cytoplasm (Fig. 1C–F), which was similar to previous results in THP-1 and J774 macrophages^8,9^. Additionally, some dividing intact bacteria were frequently observed in some host cells (Fig. 1C and D). This indicated that *S. aureus* can survive and proliferate in RAW 264.7 macrophages.

### Acid pH, not cathepsin B, decreased MP1102 and NZ2114 extracellular activities

The minimum inhibitory concentrations (MICs) of MP1102, NZ2114 and vancomycin against the *S. aureus* strains were tested at pH 7.3 and 5.0 and with cathepsin B (16 μg/ml), which is a lysosomal proteinase involved in protein degradation and found abundantly within lysosomes, to mimic the extracellular and phagolysosomal environments^23^. As shown in Table 1, the MICs for MP1102, NZ2114 and vancomycin against MRSA ATCC43300 and high virulent *S. aureus* CVCC546 at pH 7.3 were considerably lower than those in the acid environment at pH 5.0, indicating that acid pH markedly reduced the activity of the peptides. The antibacterial activities of the peptides were decreased by at least 4–16-fold under the mimic phagolysosomal environments. The MIC of vancomycin was rarely influenced by pH, which is consistent with a previous study^17^. Because acid pH 5.0 influenced the growth of MSSA ATCC25923 (Supplementary Fig. S2A), the antibacterial activities of the peptides and vancomycin markedly increased (6.8–30.3-fold decrease in MICs) (Table 1). Additionally, after treatment with cathepsin B, the MICs of MP1102 and NZ2114 (0.25–0.5 μg/ml) were not changed (Supplementary Table 4), indicating that the antibacterial activities of NZ2114 and MP1102 were not influenced by cathepsin B. This is consistent with the fact that there are no predicted cathepsin B cleavage sites in NZ2114 and MP1102 (data not shown). This result indicated that acid pH drastically reduced the activity of MP1102 and NZ2114, while cathepsin B did not affect the antibacterial activities of the peptides.

**Table 1:** The MICs of MP1102, NZ2114 and vancomycin against *S. aureus* at pH 7.3 and pH 5.0.

Drugs	pH	Net charge^a^	MICs (μg/ml)		
			MSSA ATCC25923	MRSA ATCC43300	*S. aureus* virulent CVCC546
MP1102	5.0	+5.6	<0.004	4	>8
	7.3	+2.7	0.03125	0.25	1
NZ2114	5.0	+5.6	<0.004	2	>8
	7.3	+2.7	0.0625	0.25	2
Vancomycin	5.0	ND	<0.004	1	1
	7.3	ND	0.125	1	1

^a^The charge of peptides at different pH values was analyzed by PROTEIN CALCULATOR v3.4 (C. Putnam, The Scripps Research Institute, U.S.A.) (http://protcalc.sourceforge.net/); ND: no data.

### MP1102 displayed lower cytotoxicity than NZ2114

The survival rates of RAW 264.7 macrophages were up to 100% and 85.9% when exposed to 128 and 256 μg/ml vancomycin, respectively (Supplementary Fig. S3). The survival rates of cells treated with NZ2114 were 41.8–82% at concentrations ranging from 256 to 16 μg/ml, respectively, and were higher than towards human THP-1 monocytes^17^, indicating its potent cytotoxicity against RAW 264.7 macrophages. The viability of cells treated with MP1102 was significantly lower than that of vancomycin. MP1102 toxicity was not observed up to a concentration of 32 μg/ml (survival rate >91.6%), though the survival rate was 61.2% at the 256 μg/ml concentration, indicating that MP1102 had weaker toxicity against RAW 264.7 macrophages than NZ2114.

### MP1102 and NZ2114 were internalized and distributed in the cytoplasm in RAW 264.7 macrophages

The fluorescent dye propidium iodide (PI), which can penetrate the damaged cell membrane, was used to determine the membrane penetration ability of MP1102 and NZ2114. The percentage of PI-staining in cells untreated with peptides was 0.055%, indicating the integrity of the cell membrane. After treatment with 25 and 250 μg/ml MP1102, the percentages of PI-staining in cells were 0.091% and 0.51%, respectively, indicating that MP1102 had poor cell penetrability; the percentages of PI-staining in cells treated with NZ2114 were 0.142% and 5.34%, respectively (Supplementary Fig. S4), suggesting that NZ2114 was more penetrable than MP1102.

**Figure 2:**
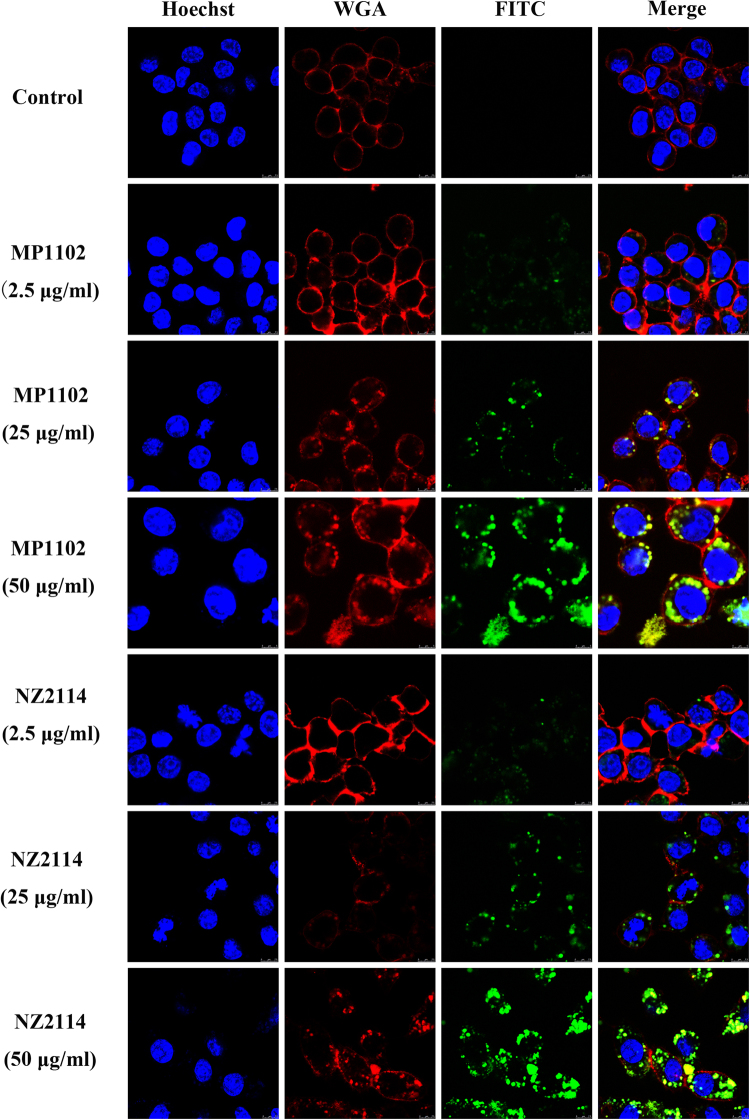
Cellular distribution of FITC-MP1102 and FITC-NZ2114 uptake in RAW 264.7 cells at 37 °C. Cells were incubated with 2.5 (10 × MIC), 25 (100 × MIC), or 50 μg/mL (200 × MIC) FITC-MP1102 and FITC-NZ2114 at 37 °C for 24 h before washing and analysis by confocal microscopy. Cell membrane and nucleus were stained with wheat germ agglutinin (WGA)-conjugated Alexa Fluor 555 (red) and Hoechst 33342 (blue), respectively. FITC-peptides inside cells displayed green fluorescence.

A prerequisite for killing intracellular *S. aureus* was the entrance of the drugs into host cells. To test whether MP1102 and NZ2114 can enter into RAW 264.7 macrophages, FITC-labeled peptides were added into cultures at different concentrations (2.5, 25, and 50 μg/ml) for 24 h. As shown in Fig. 2, the uptake of FITC-labeled peptides strongly increased with increased concentrations. Both 2.5 μg/ml and 25 μg/ml of FITC-labeled MP1102 and NZ2114 appeared to be distributed in a punctate manner inside RAW 264.7 macrophages, indicating uptake via an endocytic pathway. However, 50 μg/ml of FITC-labeled MP1102 and NZ2114 was distributed in clusters inside the cells. However, MP1102 showed a less efficient uptake than NZ2114.

**Figure 3:**
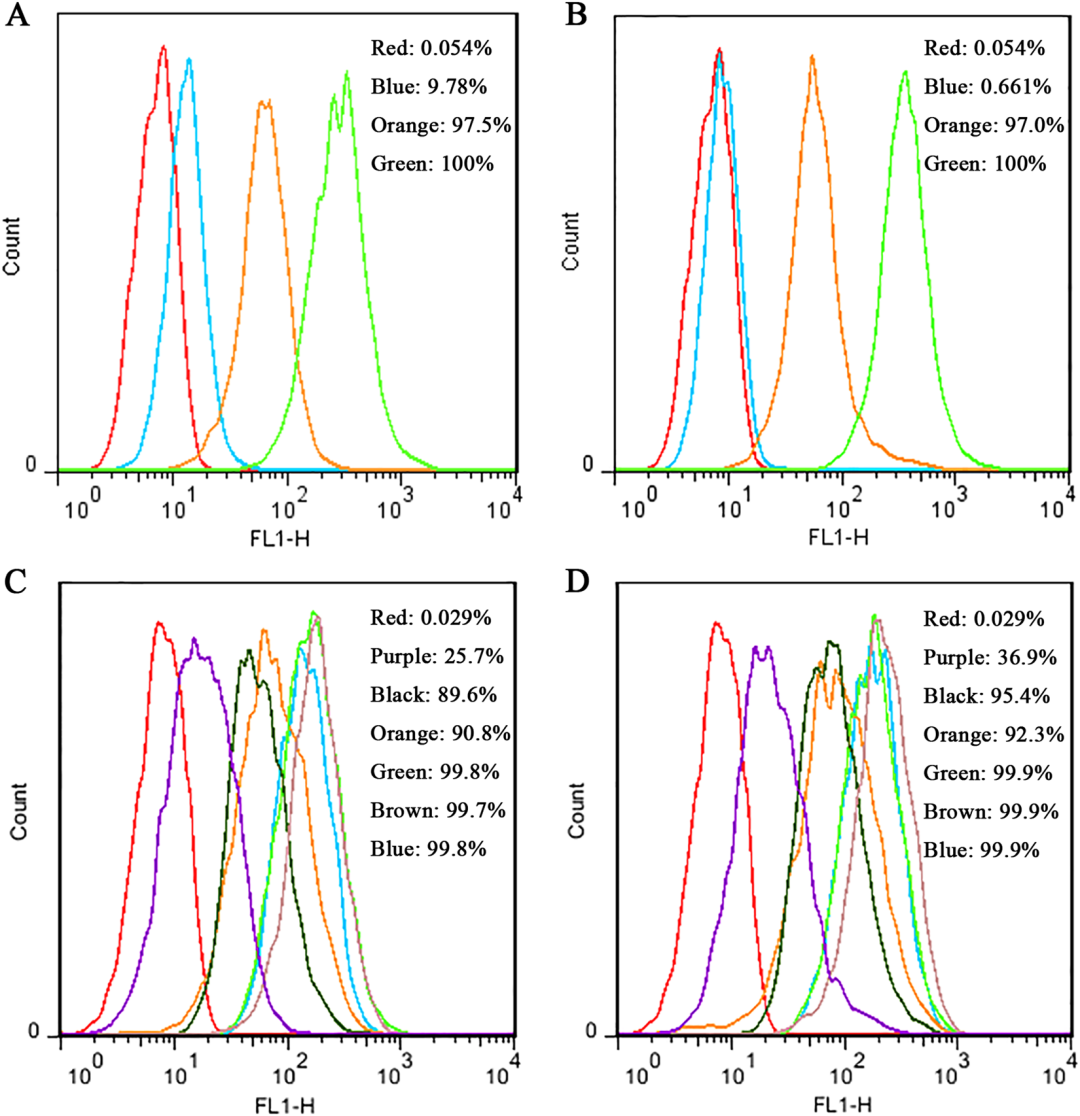
Quantification of MP1102 and NZ2114 in RAW 264.7 cells and their mechanism of cellular uptake. (**A,B**) Analysis of FITC-labeled peptide uptake by flow cytometry. The cells were incubated for 24 h with FITC-MP1102 (**A**) and FITC-NZ2114 (**B**) at 37 °C prior to washing and quantification of peptide uptake. Red line: control; blue line: 1 × MIC; orange line: 10 × MIC; green line: 100 × MIC. (**C,D**) Mechanism of cellular uptake of FITC-labeled peptide uptake. Cells were pretreated with different endocytosis inhibitors at 37 °C for 1 h prior to addition of FITC-MP1102 (**C**) and FITC-NZ2114 (**D**). The uptake of peptides was quantified by flow cytometry. Red line: control; purple line: 4 °C; black line: chlorpromazine; orange line: amiloride; green line: methyl-β-cyclodextrin (MβCD); brown line: nocodazole; blue line: 25 μg/ml peptide.

To analyze the degree of cellular uptake of the FITC-labeled peptides, the cells were further analyzed by flow cytometry to quantify the uptake efficiency. The penetrable percentages of cells treated with 1×, 10× and 100× MIC FITC-MP1102 were 9.78%, 97.5%, and 100%, respectively (Fig. 3). After treatment with 1×, 10× and 100 × MIC FITC-NZ2114, the penetrable percentages were 0.661%, 97.0%, and 100%, respectively (Fig. 3), which are much higher than that of the untreated cells (0.054%).

These data indicated that both MP1102 and NZ2114 could enter into cells via an endocytic pathway in a concentration-dependent manner and were located in the cytoplasm in RAW 264.7 macrophages.

### MP1102 and NZ2114 uptake involved clathrin-mediated endocytosis and micropinocytosis

To investigate whether the peptides enter into the cells by endocytosis, inhibitors of the endocytotic pathway and low temperature were used to pretreat the cells. As shown in Fig. 3C and D, the penetrable percentages of FITC-MP1102 and FITC-NZ2114 were 25.7% and 36.9% at 4 °C and 99.8% and 99.9% at 37 °C, indicating that low temperature had a great inhibitory effect on peptide uptake. FITC-MP1102 and FITC-NZ2114 internalization was reduced by 4.6–10.4% by chlorpromazine (an inhibitor of clathrin-mediated endocytosis) and 7.7–9.2% by amiloride (an inhibitor of macropinocytosis). However, the uptake was not inhibited by treatment with MβCD (a disruptor of lipid rafts) or nocodazole (an inhibitor of microtubule polymerization)^24–26^. These results implied that MP1102 and NZ2114 entered into cells via clathrin-mediated endocytosis and macropinocytosis.

### MP1102 and NZ2114 had extracellular bactericidal and intracellular bacteriostatic efficacy

**Figure 4:**
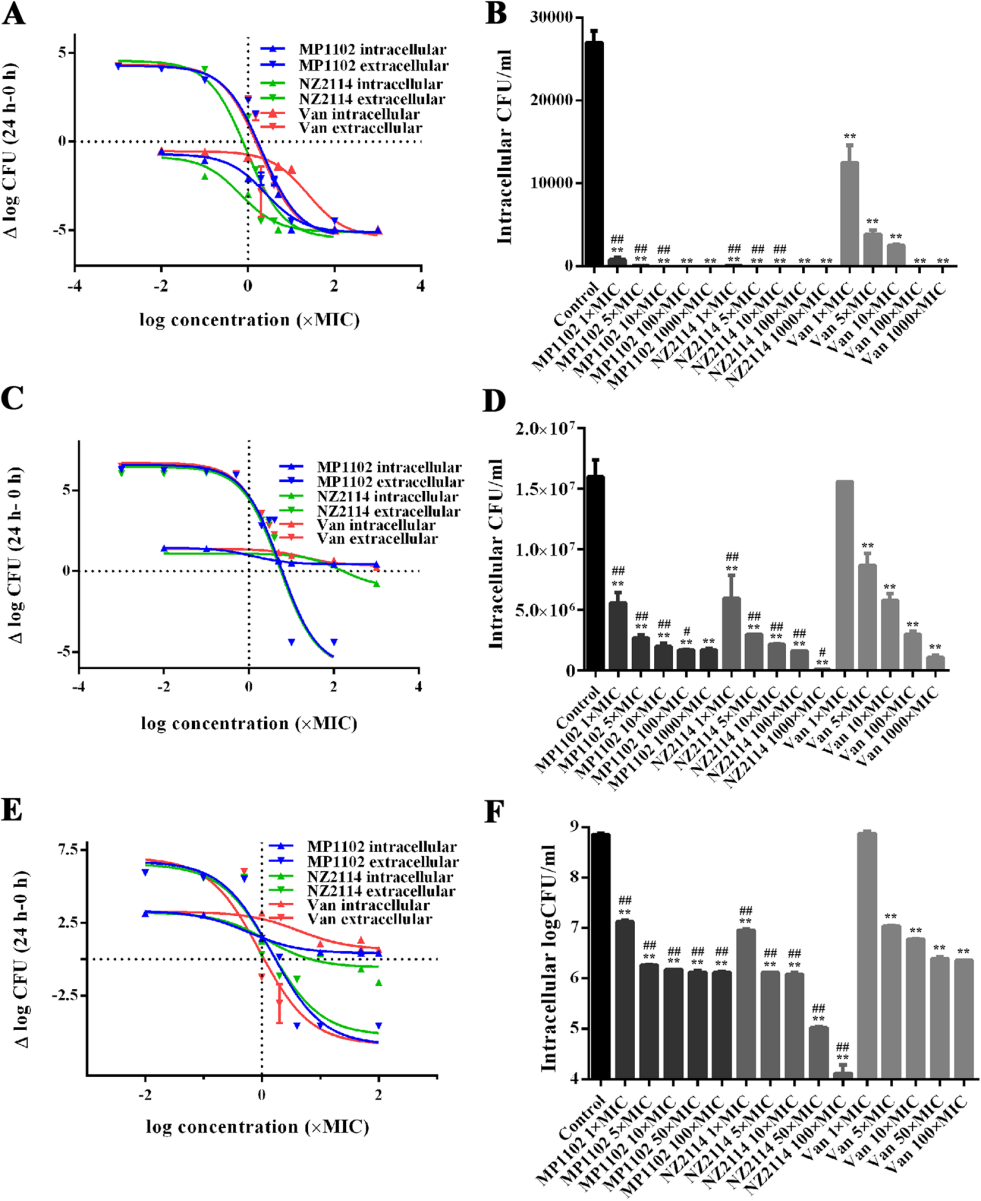
Extracellular and intracellular activity of MP1102 and NZ2114 against *S. aureus* phagocytized by RAW 264.7 cells. (**A,C,E**) Concentration-response curves of MP1102, NZ2114 and vancomycin against *S. aureus*. The ordinate showed bacterial reduction (log CFU from the initial inoculum) per ml of broth in RAW 264.7 cells after 24 h of incubation with peptides compared to the original inoculum. (**B,D,F**) Intracellular activity of MP1102, NZ2114 and vancomycin against *S. aureus* compared to the PBS treated group. (**A,B**) MSSA ATCC25923; (**C,D**) MRSA *S. aureus* ATCC43300; (**E,F**) virulent *S. aureus* CVCC546. Van, vancomycin. Statistical analyses were performed using IBM SPSS Statistics 21.0. The analyses were measured by one-way ANOVA, with Duncan’s multiple comparisons test. A probability value of <0.05 was considered significant. (*) Indicates the significance between control and treatment groups. *P < 0.05; **P < 0.01. (#) Indicates significance between the AMPs and Van groups with same drug dose. ^#^*P* < 0.05; ^##^*P* < 0.01. Values represent as the mean ± standard errors (SEM) of three independent experiments performed in triplicate.

The effect of peptide concentrations (0.001–100 × MIC) on extracellular activity was determined against three *S. aureus* strains over a 24-h period. As shown in Fig. 4 and Table 2, MP1102, NZ2114 and vancomycin exhibited distinct bactericidal efficacy. The maximal efficacy (E_max_) values for all the strains were calculated to be >5-log decrease in CFU compared to the initial inoculum after 24 h. Similar to vancomycin, both MP1102 and NZ2114 showed a concentration-dependent killing manner. Against MRSA ATCC43300, MSSA ATCC25923 and high virulent *S. aureus* CVCC546, the peptides and vancomycin displayed similar relative potencies (50% effective concentrations (EC_50_)) at approximately 5 times and 1–2 times the MICs, respectively. The static concentrations (C_s_) varied from 0.76 to 6.02 times the MICs, and MRSA ATCC43300 had the strongest ability to resist high MIC-fold concentration drugs.

**Table 2:** Maximal relative efficacy (E_max_) and C_s_ of MP1102, NZ2114, and vancomycin, as determined from analysis of the data presented in Fig. 4.

Strains	Antimicrobials	Intracellular				Extracellular			
		E_max_^a^ (log CFU, 95% CI)	EC_50_^b^	C_s_^c^	R^2^	E_max_^a^ (log CFU, 95% CI)	EC_50_^b^	C_s_^c^	R^2^
MSSA ATCC25923	MP1102	−4.41 (−5.08 to −3.74)	2.55 (1.31 to 4.93)	—	0.930	−5.37 (−6.41 to −4.33)	2.10 (1.41 to 3.14)	~1.66	0.950
	NZ2114	−4.26 (−4.85 to −3.66)	0.67 (0.34 to 1.34)	—	0.958	−5.47 (−6.57 to −4.36)	0.91 (0.54 to 1.53)	~0.76	0.917
	Vancomycin	−4.85 (−5.37 to −4.34)	24.29 (14.66 to 40.25)	—	0.976	−5.37 (−6.64 to −4.09)	1.87 (1.14 to 3.08)	~1.48	0.898
MRSA ATCC43300	MP1102	0.42 (0.37 to 0.47)	1.25 (0.79 to 1.96)	—	0.993	−5.95 (−7.77 to −4.14)	5.52 (3.45 to 8.83)	~6.02	0.904
	NZ2114	−1.02 (−1.86 to −0.19)	163.17 (37.33 to 713.24)	~165.96	0.833	−6.05 (−7.63 to −4.47)	5.39 (3.60 to 8.09)	~5.75	0.918
	Vancomycin	0.43 (0.38 to 0.47)	1.10 (0.82 to 1.48)	—	0.990	−5.89 (−7.61 to −4.18)	5.40 (3.45 to 8.47)	~5.88	0.901
Virulent *S. aureus* CVCC546	MP1102	0.41 (0.31 to 0.50)	0.59 (0.44 to 0.79)	—	0.993	−5.88 (−7.16 to −4.60)	1.49 (0.94 to 2.36)	~1.70	0.933
	NZ2114	−1.07 (−1.65 to −0.49)	2.53 (1.13 to 5.64)	~7.16	0.934	−5.23 (−6.39 to −4.08)	1.39 (0.88 to 2.17)	~1.74	0.935
	Vancomycin	0.46 (0.10 to 0.82)	3.53 (1.81 to 6.87)	—	0.951	−5.85 (−7.48 to −4.22)	0.92 (0.48 to 1.74)	~1.10	0.880

^a^Maximal relative efficacy (decrease in log CFU at 24 h from the corresponding original inoculum), as extrapolated for antibiotic concentrations at infinitely high concentrations. CI, confidence interval; ^b^The concentration (in multiples of the MIC) causing a 50% reduction between the minimal and maximal values, as obtained from the Hill equation; ^c^The concentration (in multiples of the MIC) resulting in no apparent bacterial growth (the number of CFU was identical to that of the original inoculum), as determined by the graphical interpolation.

The intracellular activities of the peptides and vancomycin were also tested over a wide range of concentrations (0.01 to 100 or 1000 × MIC) for 24 h against *S. aureus* phagocytosed by RAW 264.7 macrophages. The intracellular E_max_ values for MP1102, NZ2114 and vancomycin were sharply reduced compared to the extracellular values (Table 2). The E_max_ values for MP1102, NZ2114 and vancomycin against MSSA ATCC25923 were −4.41, −4.26, and −4.85 log CFU, respectively, indicating their intracellular bactericidal activities. In contrast, the EC_50_ values were significantly different for MP1102 and NZ2114 against MSSA ATCC25923 and high virulent *S. aureus* CVCC546, which showed no significant changes compared to their corresponding values against extracellular bacteria. In contrast, a marked decrease (161-fold) in potency was noted for NZ2114 against MRSA ATCC43300. For MRSA ATCC43300 and virulent *S. aureus* CVCC546, only NZ2114 could maintain the intracellular CFU; C_s_ is identical to the original inoculum at the concentrations at 165.96 (ATCC43300) and 7.16 (CVCC546) times the MIC. However, the C_s_ of MP1102 and vancomycin could not be obtained, as their resistant rates for the highest test concentrations compared to the untreated group were up to 91.19% and 93.03% for MRSA ATCC43300 and 99.81% and 99.68% for high virulent CVCC546 (Fig. 4), respectively. For MSSA ATCC25923, the CFU number of the untreated group was lower than the original inoculum. No bacterial colonies were observed in the treated groups at high MIC fold concentrations. Notably, the intracellular antibacterial activities of MP1102 and NZ2114 were higher than that of vancomycin, but significantly lower than the extracellular values (Table 2). These results suggested that MP1102 and NZ2114 only had bacteriostatic effects against intracellular MRSA ATCC43300 and high virulent *S. aureus* CVCC546.

### MP1102 and NZ2114 regulated cytokines in RAW 264.7 macrophages

**Figure 5:**
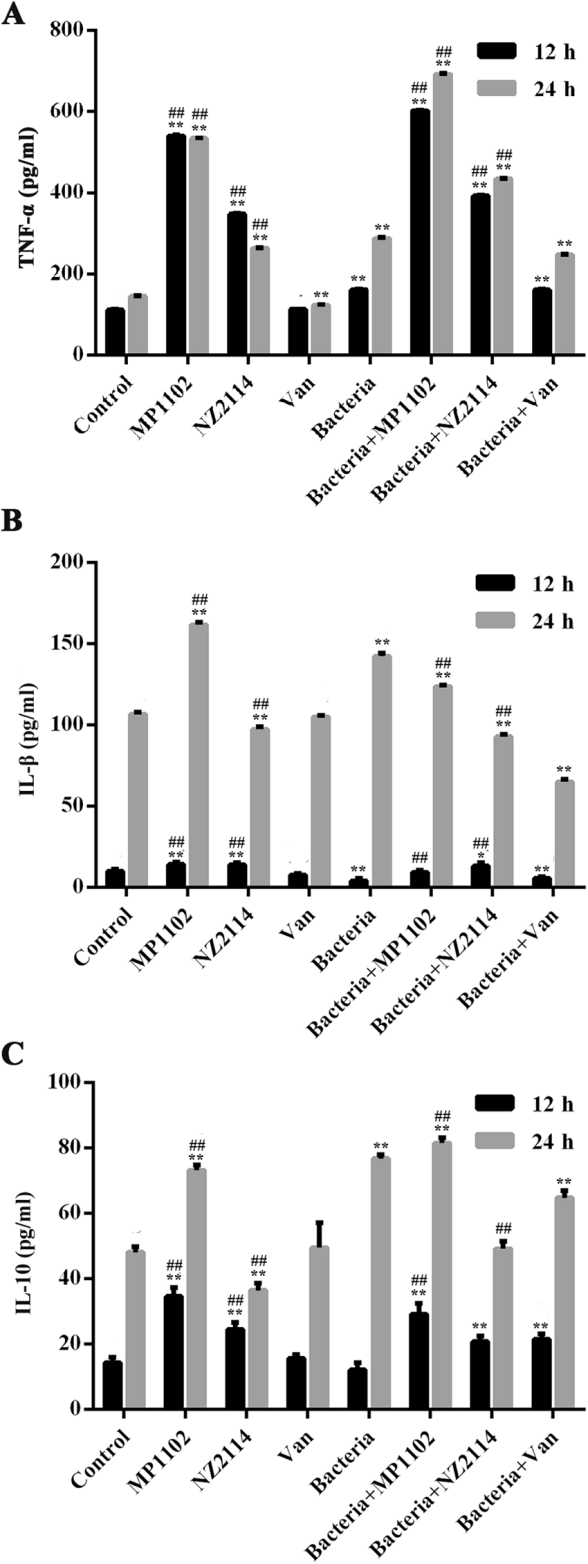
Effect of MP1102 and NZ2114 on TNF-α, IL-1β and IL-10 in RAW 264.7 Cells. The concentrations of TNF-α (**A**), IL-1β (**B**) and IL-10 (**C**) were measured using enzyme linked immunosorbent assay (ELISA) after 12 h and 24 h of sample treatment, respectively. Van, vancomycin. Statistical analyses were performed using IBM SPSS Statistics 21.0. The analyses were measured by one-way ANOVA, with Duncan’s multiple comparisons test. A probability value of <0.05 was considered significant. (*) Indicates the significance between control and treatment groups. *P < 0.05; **P < 0.01. (#) Indicates significance between the AMPs and Van groups with same drug dose. ^#^*P* < 0.05; ^##^*P* < 0.01. Values represent as the mean ± SEM of three independent experiments performed in triplicate.

To further explore the effects of the peptide on cytokines, normal RAW 264.7 macrophages and *S. aureus-*challenged cells were treated with peptides or antibiotic for 12 h or 24 h. As shown in Fig. 5, the cytokine levels of tumor necrosis factor (TNF)-α, interleukin (IL)−1β and IL-10 in the control groups at 12 h were 111.153 ± 1.242, 9.806 ± 1.502, and 14.187 ± 1.667 pg/ml, respectively. The cytokine production levels of TNF-α, IL-1β, and IL-10 in the cell-free culture supernatants were significantly enhanced in response to *S. aureus* at 24 h after infection. Similar to vancomycin, both MP1102 and NZ2114 significantly suppressed the production of IL-1β at 24 h (18.7–49.5 pg/ml) after infection (Fig. 5B). However, the two peptides and vancomycin promoted the secretion of TNF-α (0.4–341.3 pg/ml), IL-1β (1.6–9.2 pg/ml) and IL-10 (7.1–9.5 pg/ml) at 12 h and TNF-α (146.1–403.6 pg/ml, exception for vancomycin) at 24 h (Fig. 5B and C). Additionally, 24 h after treatment with NZ2114 and vancomycin, IL-10 levels had decreased by 27.6 pg/ml and 12.0 pg/ml, respectively, but in contrast to that of MP1102 (4.8 pg/ml). This result indicated that MP1102 and NZ2114 more potently regulate immune system macrophages than vancomycin.

### MP1102 and NZ2114 had potent intracellular therapeutic efficacy *in vivo*

**Figure 6:**
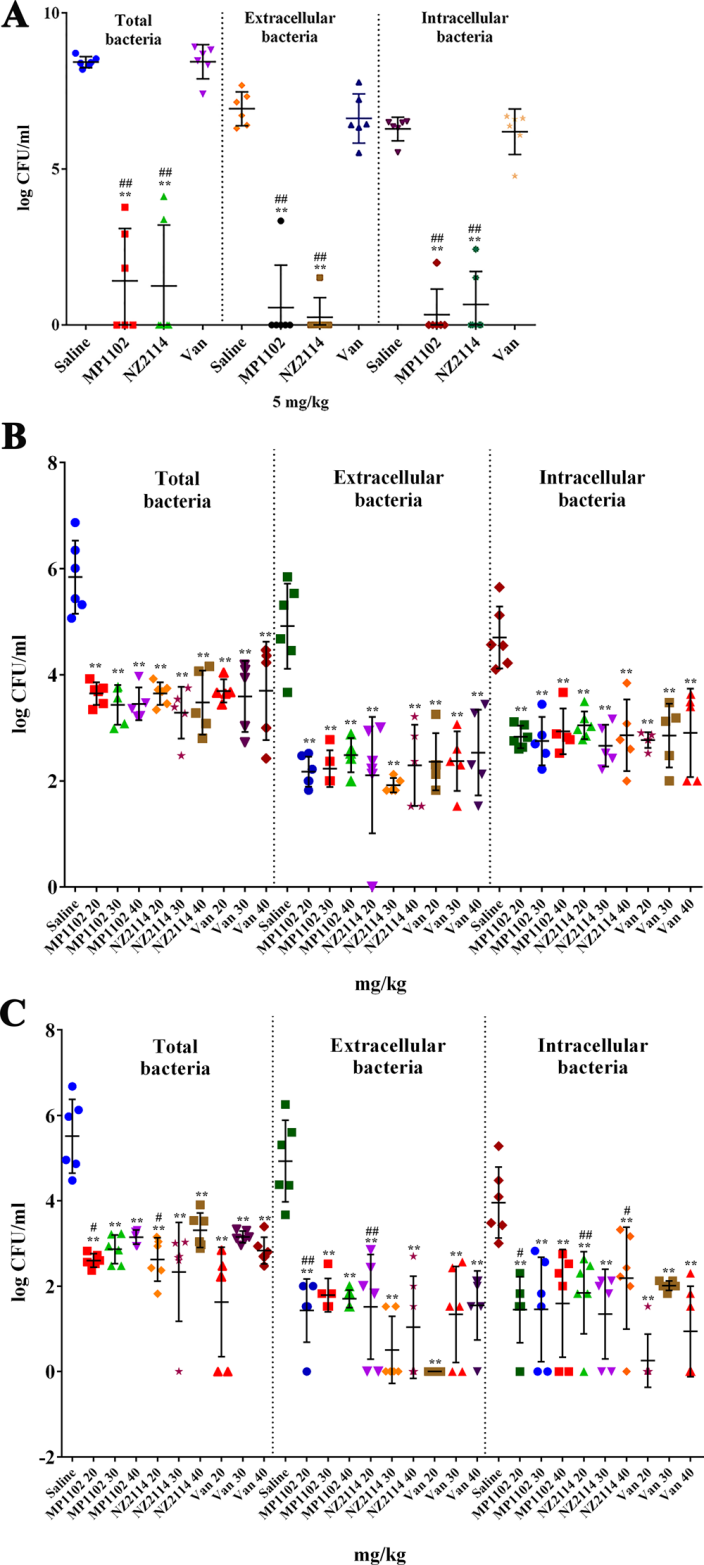
Extracellular and intracellular activity of MP1102, NZ2114 and vancomycin in the murine peritonitis model. The mice were peritoneally injected with MSSA ATCC25923 (**A**), MRSA ATCC43300 (**B**) and virulent *S. aureus* CVCC546 (**C**). Bacterial loads in total peritoneal fluid, extracellular and intracellular after 24 h of treatment were counted. Each point represents data from a single mouse. Mean values are presented, n = 5 or 6. Error bars indicate ± SEM. Van, vancomycin. Statistical analyses were performed using IBM SPSS Statistics 21.0. The analyses were measured by one-way ANOVA, with Duncan’s multiple comparisons test. A probability value of ˂ 0.05 was considered significant. (*) Indicates the significance between control and treatment groups. *P < 0.05; **P < 0.01. (#) Indicates significance between the AMPs and Van groups with same drug dose. ^#^*P* < 0.05; ^##^*P* < 0.01.

To determine the *in vivo* intracellular antibacterial activity, MP1102 and NZ2114 were tested in a mouse intraperitoneal injection model. Mice challenged with *S. aureus* were treated with MP1102, NZ2114 or vancomycin and bacterial burden was monitored in the total peritoneal fluid and extracellular and intracellular cells. For MSSA ATCC25923, the 20 mg/kg and 10 mg/kg dose of MP1102 and NZ2114 treatments were so effective that no colonies were observed on the plates (data not shown). When the dose was reduced to 5 mg/kg, the MP1102 and NZ2114 groups showed a sharp decrease in CFU for the total (7.01 and 7.18 log reduction, respectively), extracellular (6.37 and 6.68 log reduction, respectively) and intracellular (5.95 and 5.63 log reduction, respectively) bacteria, which may be related to the improved alkalinization of the lysosomes by the peptide molecules^27^. However, no significant effects were observed in the vancomycin group (Fig. 6A). For MRSA ATCC43300, 20–40 mg/kg MP1102 and NZ2114 showed an approximately 2.1–2.6 log reduction for total, 2.7–3.0 log reduction for extracellular and 1.7–2.1 log for intracellular bacterial compared to the untreated controls (Fig. 6B) and similar to vancomycin. However, different antibacterial effects were observed for the treatment of high virulent *S. aureus* CVCC546 (Fig. 6C). The burden of bacteria was significantly reduced in the total (3.9 log reduction), extracellular (4.9 log reduction) and intracellular (3.7 log reduction) bacteria in the 20 mg/kg vancomycin-treated group. Comparably, NZ2114 and MP1102 led to relatively lower efficiencies, with a log CFU decrease of 2.2–3.2, 3.1–4.4 and 1.8–2.6 for total, intracellular, and extracellular bacteria, respectively. These data indicated that MP1102 and NZ2114 had almost equal intracellular therapeutic efficacy *in vivo* to the extracellular values, which is independent on doses of peptides or antibiotic.

## Discussion

*S. aureus* has the ability to invade and survive inside phagocytes and other cells, thus contributing to the persistence and recurrence of its infections^5,28^. A previous study demonstrated that *S. aureus* ATCC25923 and ATCC43300 can enter into and survive in THP-1 cells^9^. In our study, *S. aureus* ATCC25923, ATCC43300 and CVCC546 were chosen as a representative of fully susceptible, resistant and high virulent strains to investigate whether MP1102 and NZ2114 can enter into RAW 264.7 macrophages and kill intracellular bacteria.

Our study showed that mimic phagolysosomal environments had an obvious effect on the growth of MSSA ATCC25923, but not for MRSA ATCC43300 and high virulent *S. aureus* CVCC546 (Supplementary Fig. S2), indicating that they are more resistant to phagosomal acidification and are more dangerous intracellular strains. The antibacterial activities of both NZ2114 and MP1102, which have higher MICs, were impaired by the acidic pH in macrophages (Table 1), which is similar to many antibiotics such as aminoglycosides and macrolides (especially azithromycin and telithromycin)^8,29–31^. This may be related to the larger ionization of drugs at acidic pH than at neutral pH, which may impair their transport into bacteria^9^; in contrast, low pH may change the electrostatic potential of bacterial cell membranes, which can affect the uptake of drugs into the bacterial cells and their antibacterial activities^30^. The activity of vancomycin was largely unaffected by low pH, which was consistent with a previous study^17^. This discrepancy may be related to following different interactions formed between Lipid II and drugs: i) the pyrophosphate moiety forms hydrogen bonds to F2, G3, C4, and C27 in the peptides and ii) D-γ -glutamate from Lipid II forms a salt bridge with the peptide N termini and the His18 side-chain^18,32^. However, the primary interactions between vancomycin and Lipid II involve the D-alanyl-D-alanine (D-ala-D-ala) part of the Lipid II pentapeptide^32^. Therefore, we deduced that the interactions between the plectasin derivates MP1102 and NZ2114 and Lipid II were weaker than that of vancomycin and Lipid II. Further studies are needed to determine the peptides’ detailed mechanism for their good intracellular activity.

This study is the first evaluation of the intracellular anti-staphylococcal properties of the NZ2114 derivative MP1102 using both *in vitro* and in *vivo* methods. Plectasin, NZ2114 and vancomycin have been shown to have anti-intracellular *S. aureus* efficiency in human THP-1 macrophages^14,17^. As a corresponding *in vivo* mouse model, RAW 264.7 macrophages were chosen to evaluate *in vitro* intracellular activity in this study. MP1102, NZ2114 and vancomycin exhibited extracellular bactericidal activities *in vitro* against *S. aureus* (>5 log kill) (Table 2) in the RAW 264.7 macrophages, which was similar to that of NZ2114 and plectasin in previous studies^14,17^. The *in vitro* intracellular activity of NZ2114 towards *S. aureus* susceptible or resistant strains in RAW 264.7 macrophages (E_max_:−4.26– −1.02; C_s_: 165.96) in our study was far superior to that of NZ2114 (E_max_:−1.51– −0.93; C_s_: 0.8–1.1) and plectasin (E_max_:−1.4– −1.0; C_s_: 0.6) in THP-1 cells in previous studies^14,17^. The relative E_max_ values of all the drugs against intracellular *S. aureus* resistant and high virulent strains were largely reduced by 3.9–13.3-fold due to low pH, with values of approximately 1 log CFU for NZ2114 compared to values of more than 5.2 log CFU for extracellular bacteria (Table 2 and Fig. 4), which were higher than those of NZ2114 (0.93 and 4.58 log CFU) against MRSA in THP-1 cells^17^. The same phenomenon has also been observed with other antibiotics, such as macrolides (such as telithromycin), aminoglycosides (such as gentamicin) and AMPs (such as plectasin and NZ2114) in Vero cells, THP-1 and J774 macrophages, respectively, in intracellular anti-staphylococcal studies^8–10,14,17,29–31^. Previous studies have demonstrated that the combination of antibiotics (such as aminoglycosides and doxycycline) with basic lysosomal alkalinization agents (such as chloroquine, methylamine, and ammonium chloride) can increase their intracellular activity against *Coxiella burnetii* and *S. aureus* in P388D1 cells^27,33–35^, indicating that phagolysosomal alkalization is very helpful for improving intracellular activity^9,35^. However, it also has been reported that these drugs may be sequestered in lysosomes and bind to phospholipids via electrostatic interactions, which leads to reduced intracellular activity^8^. Additionally, the *in vitro* intracellular activity of NZ2114 (with E_max_ of −1.02– −1.07 log CFU) against MRSA ATCC43300 and virulent *S. aureus* CVCC546 was more potent than those of MP1102 (with E_max_ of 0.41–0.42 log CFU) and vancomycin (with E_max_ of 0.43–0.46 log CFU) (Table 2). Similar to plectasin (with E_max_ of −1.0– −1.4 log CFU), in this work, NZ2114 also showed higher relative efficacy than azithromycin (with E_max_ of −0.5 log CFU), oxacillin (with E_max_ of −0.68), linezolid, and imipenem (with E_max_ of −0.9), which are commonly used to combat virulent staphylococcal strains^14,31,36,37^.

Due to several limitations (including protein binding conditions and differences in the pharmacokinetics) in the *in vitro* macrophage intracellular model^17^, an *in vivo* mouse peritonitis model was also examined to further evaluate the intracellular efficiency of MP1102 and NZ2114. The intracellular bacterial killing efficacy of MP1102 and NZ2114 was decreased *in vivo* compared to extracellular killing, though the difference was less than the *in vitro* model, which may be related to the complex *in vivo* environment weakening the antibacterial efficiency and the eradication of extracellular bacteria, which reduces the amount of bacteria entering into cells^14^. Additionally, there are several factors affecting the intracellular activity of drugs, such as the balance between influx and efflux, metabolism, and protein binding, which determine the intracellular concentration of free active drugs^38^. Though the intracellular activity was proven to have a remote correlation with the actual extracellular drug concentration and even the degree of cellular concentration, it does not mean that the cellular accumulation of drugs was irrelevant to their effective activity. The prerequisite of anti-intracellular bacterial drugs is that drugs can reach their targets and come into contact with the bacteria^38^. It has been demonstrated that vancomycin exhibited a slow uptake and accumulation in macrophages (up to 8-fold at 24 h)^39^. Likewise, in this study, MP1102 and NZ2114 could enter into RAW 264.7 macrophages in a dose-dependent manner and were distributed in the cytoplasm (Fig. 2), which was similar to hLF, R9 and the TAT peptide^40,41^.

**Figure 7:**
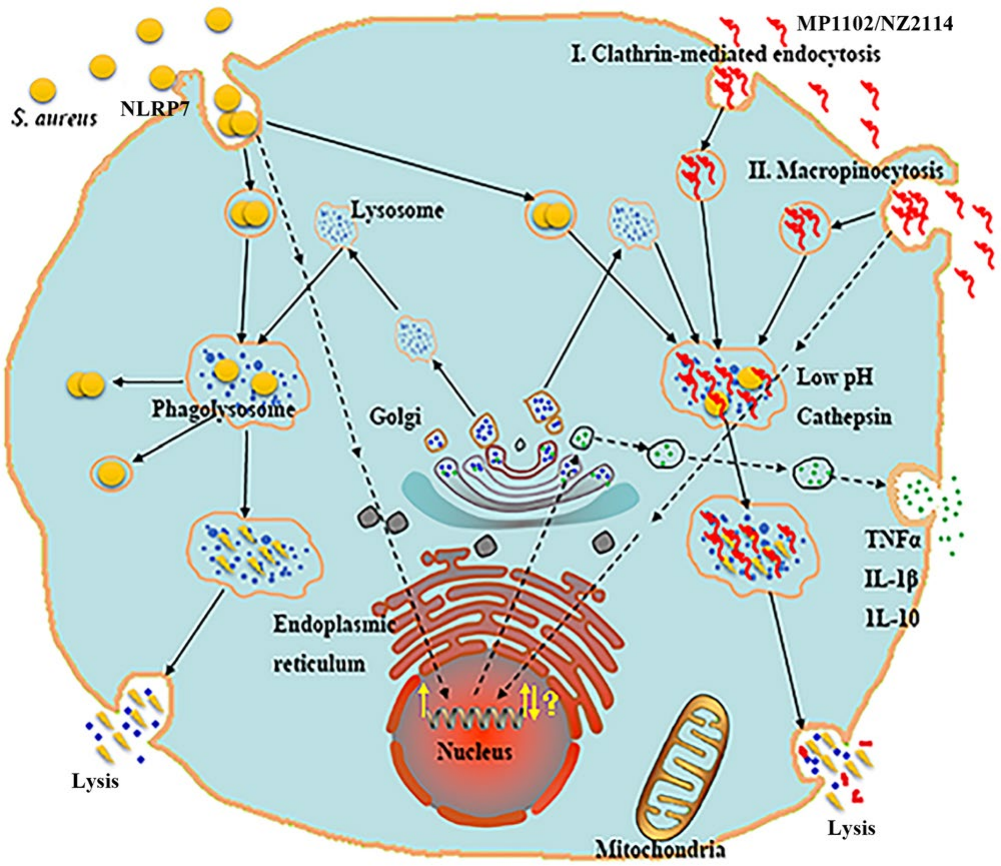
Mechanism of MP1102 and NZ2114 uptake into RAW 264.7 cells and their intracellular activity and immunomodulatory properties. After invaded into RAW 264.7 cells, *S. aureus* are disinfected by phagolysosomes or survive and grow within endosomes or in the cytoplasm after phagosomal escape^5,58^. Both MP1102 and NZ2114 were internalized into macrophages by clathrin-mediated endocytosis (I) and macropinocytosis (II) in an energy- and temperature-dependent manner and ultimately entered into lysosomes^7,44^. MP1102 and NZ2114 exhibited potent efficacy against intracellular forms of *S. aureus* in RAW 264.7 cells and in mice. Additionally, the production levels of TNF-α, IL-1β and IL-10 in RAW 264.7 cells were regulated by MP1102 and NZ2114. The (NOD)-like receptor (NLR) containing a PYRIN domain (PYD) 7 (NLRP7) can especially recognize acylated lipopeptides of *S. aureus* in macrophages, which may promote caspase-1 activation and IL-1β maturation^44,52^.

Drug internalization mechanisms are usually assessed using different endocytosis inhibitors that act by selectively blocking specific endocytosis pathways^24^. In this study, the entrance of MP1102 and NZ2114 was blocked by low temperature, chlorpromazine and amiloride (Fig. 3C and D). MβCD and nocodazole did not affect the uptake of FITC-labeled MP1102 and NZ2114. This result indicated that clathrin-mediated endocytosis and macropinocytosis are the major routes of MP1102 and NZ2114 internalization in an energy-and temperature-dependent manner (Fig. 7), which was similar to TAT, α1H and α2H cell-penetrating peptides^42,43^. Clathrin-mediated endocytosis and micropinocytosis suggest that the degradative route follows from early endosome-like organelles to late endosome-like organelles, and ultimately to lysosomes (Fig. 7)^44^.

Previous studies have demonstrated that the cecropin-melittin hybrid peptides CEME and PPY1 significantly inhibited the release of proinflammatory cytokines in LPS-stimulated macrophages in a dose-dependent manner^45,46^. In this study, our data revealed that MP1102 and NZ2114 elevated TNF-α, IL-1β and IL-10 levels in *S. aureus*-infected RAW 264.7 macrophages at 12 h and inhibited IL-1β levels at 24 h (Fig. 5). It should be noted that this inhibitory effect was not due to the cytotoxic activity of the two peptides, as cell viability was not obviously affected by treatment with MP1102 and NZ2114 at lower than 32 μg/ml (Supplementary Fig. S3). In general, both MP1102 and NZ2114 not only have antibacterial functions but also have immunomodulatory properties (Fig. 7), especially MP1102, which was similar to previous studies wherein human α- and β-defensins and the fungal plectasin were shown to be immune-related potassium voltage-gated channel subfamily A member 3 (Kv1.3) channel inhibitors, and further suppressed IL-2 production in T cells^47–51^. Due to their similar cysteine-stabilized alpha-beta (CSαβ) structure to β-defensin and plectasin, MP1102 and NZ2114 may inhibit the Kv1.3 channel by binding to the channel extracellular pore region, which may confer to a novel Kv1.3 channel-mediated immunomodulatory function for the peptides^48^. This hypothesis needs to be further investigated. Moreover, the intracellular nucleotide-binding oligomerization domain NLR can specifically recognize acylated lipopeptides of *S. aureus* in macrophages and promote caspase-1 activation and IL-1β maturation (Fig. 7)^44,52^.

In conclusion, the antibacterial activities of MP1102 and NZ2114, which also displayed low cytotoxicity, was reduced by an acidic intracellular environment, but not by cathepsin B. MP1102 and NZ2114 could enter into RAW 264.7 macrophages through endocytosis and macropinocytosis in a dose-dependent manner and were distributed in the cytoplasm in cells. The intracellular activities of MP1102 and NZ2114 are inferior to the extracellular activities, which is similar or superior to vancomycin against methicillin-susceptible, resistant and high virulent *S. aureus in vitro* and *in vivo*. Both MP1102 and NZ2114 also regulated cytokines in *S. aureus*-infected macrophages. Together, these findings suggest that MP1102 and NZ2114 have potential for treating infections caused by intracellular *S. aureus* and reducing chronic and recurrent infections.

## Materials and Methods

### Bacterial strains, cell lines, mice and growth conditions

The test strains, including *S. aureus* ATCC25923 (MSSA), ATCC43300 (MRSA) and CVCC546 (clinical high virulent), were purchased from the China Veterinary Culture Collection Center (Beijing, China). All strains were cultured in Mueller – Hinton broth (MHB) at 37 °C and their growth curves, antimicrobial susceptibility and SCC*mec* or *spa* typing were measured as described in detail in the supplemental information. RAW 264.7 macrophages were obtained from Peking Union Medical College and grown in DMEM with 10% fetal bovine serum (FBS) (Invitrogen Trading (Shanghai) Co., Ltd.) supplemented with 5% penicillin/streptomycin (Invitrogen Trading (Shanghai) Co., Ltd.). Cells were cultivated at 37 °C in 5% carbon dioxide. Six-week-old specific pathogen-free female ICR mice were purchased from Vital River Laboratories (VRL, Beijing).

### Intracellular macrophage infections with *S. aureus*

RAW 264.7 macrophages were infected with MSSA ATCC25923, MRSA ATCC43300 and high virulent *S. aureus* CVCC546 as described previously^53^. Phagocytosis assay was performed for 0.5 h at 37 °C at a MOI of 100:1 (bacteria to macrophages), resulting in >85% of macrophages engulfing at least one bacterium. Non-phagocytosed bacteria were removed from the outside of cells by gentamicin or lysostaphin. The RAW 264.7 macrophages were then rinsed twice with PBS, re-suspended, fixed in 2.5% glutaraldehyde, and osmicated in 1% OsO4. After washing, the cells were dehydrated with a series of graded ethyl alcohols (50%, 70%, 85%, 95%, and 100%). The samples were immersed in the mixtures of absolute ethyl alcohol and resin (1:1) for 2 h, followed by incubation with epoxy resin overnight. After being embedded into capsules, the samples were polymerized at 45 °C for 3 h and at 65 °C for 24 h. Thin sections were prepared using an ultramicrotome, stained with 1% uranyl acetate, and visualized with a JEM1400 (JEDL, Tokyo, Japan)^54^.

### Effect of pH and cathepsin B on MP1102 and NZ2114 extracellular activities

The cleavable cathepsin sites in the peptides were predicted by the SitePrediction website (http://www.dmbr.ugent.be/prx/bioit2-public/SitePrediction/)^55^. The MICs of MP1102 and NZ2114 against MRSA ATCC43300, MSSA ATCC25923 and clinical high virulent *S. aureus* CVCC546 were determined in MHB adjusted to a specific pH value 5.0 to mimic the pH conditions of endosomes and lysosomes by the addition of 2 M HCl and without adjustment at a standard pH value 7.3^8,29^.

To determine the effect of phagolysosomal enzyme-cathepsin B on the peptides’ antibacterial activities, 320 μg/ml MP1102 and NZ2114 were incubated for 1 h in a 16 μg/ml enzyme solution (20 mM sodium acetate, 1 mM EDTA, and 5 mM L-cysteine, pH 5.0) at 37 °C^55^. The MICs of the peptides before and after incubation were examined against MRSA ATCC43300 as described previously^56^.

### Cytotoxicity of MP1102 and NZ2114 toward RAW 264.7 macrophages

The cytotoxicity of MP1102 and NZ2114 was tested by a colorimetric 3-(4, 5-dimethyl-2-thiazolyl)-2, 5-diphenyl-2-H-tetrazolium bromide (MTT) assay^54^. Cells were added into 96-well microtiter plates (2.5 × 10^4^ cells/well) and incubated for 24 h. After another 24 h incubation with MP1102 and NZ2114 (1 to 256 μg/ml), MTT solution was added and incubated for 4 h. Followed the removing of the MTT, dimethyl sulfoxide (DMSO) (150 μl/well) was added and the absorbance was measured at 570 nm. Untreated cells were used as a control. The cell survival rate was calculated using the following formula: survival rate (%) = Abs_570 nm_ of treated sample / Abs_570 nm_ of control × 100. The cytotoxicity detection was repeated in triplicate.

### Translocation, subcellular distribution and quantification of MP1102 and NZ2114 uptake into RAW 264.7 macrophages

The RAW 264.7 macrophages were seeded into 12-well plates (1.875 × 10^5^ cells/well) and incubated for 24 h. Then, 25 μg/ml and 250 μg/ml of MP1102 or NZ2114 solutions were added to the cells and incubated at 37 °C for an additional 24 h. After washing twice with PBS, the cells were then stained with 50 μg/ml PI for 10 min and analyzed using a FACS Calibur Flow Cytometer (BD, USA)^46^.

Next, RAW 264.7 macrophages were seeded in a confocal dish at a concentration of 1 × 10^4^ cells/dish and incubated for 24 h. FITC-labeled MP1102 and NZ2114 were added to the cells to final concentrations of 2.5 (10 × MIC for MRSA ATCC43300), 25 (100 × MIC) and 50 μg/ml (200 × MIC) and incubated for 24 h at 37 °C. After the incubation, the cells were then rinsed with PBS and stained with WGA-conjugated Alexa Fluor 555 (5 μg/ml, Invitrogen) and Hoechst 33342 (5 μg/ml, Invitrogen) for membrane and nuclear staining, respectively, for 10 min before confocal microscopy^41,57^.

To quantify peptide uptake, RAW 264.7 macrophages were grown in 6-well plates (2.5 × 10^5^ cells/ml, 1.5 ml/well) for 24 h. Then, the cells were similarly treated with FITC-MP1102 and FITC-NZ2114 (0.25, 2.5 and 25 μg/ml) for 24 h and washed with PBS. To avoid the influence of membrane-bound FITC-labeled peptides, the cells were incubated with 0.04% trypan blue in PBS for 15 min. The fluorescence intensity was analyzed using a FACS Calibur Flow Cytometer (BD, USA)^57^.

### Mechanism of MP1102 and NZ2114 cellular uptake

To determine the effects of endocytosis inhibitors on peptide uptake, RAW 264.7 macrophages were pretreated with 3 mM amiloride, 5 mM MβCD, 20 µM nocodazole, and 6 μg/ml chlorpromazine for 1 h at 37 °C prior to the addition of 25 μg/ml FITC-MP1102 and FITC-NZ2114 for 6 h at 37 °C. Cells treated with FITC-labeled peptides at 37 °C for 6 h were used as a positive control. To inhibit the endocytic machinery, cells were treated with FITC-labeled peptides at 4 °C for 6 h. Finally, the cells were mixed with 0.04% trypan blue and the FITC-labeled peptides within cells were measured using flow cytometry^42^.

### *In vitro* efficacy of MP1102 and NZ2114 towards the extracellular and intracellular forms of *S. aureus*

Dose-response curves for the extracellular activities of the peptides were performed as previously described with some modifications^2,14,56,57^. For extracellular activity, MSSA ATCC25923, MRSA ATCC43300 and high virulent *S. aureus* CVCC546 cells were adjusted to a density of 10^5^ CFU/ml. After 24 h of incubation with different concentrations of the peptides (0.001–100 × MIC), the number of viable bacteria was determined by colony counting.

For intracellular activity, RAW 264.7 macrophages (2.5 × 10^5^ cells/ml) were seeded into a 6-well plate (1.5 ml/well) and cultured for 24 h in DMEM with 10% FBS (without antibiotics). Meanwhile, mid-log phase *S. aureus* strains (MSSA ATCC25923, MRSA ATCC43300 and high virulent CVCC546) were collected by centrifugation, diluted to a concentration of 2.5 × 10^7^ CFU/ml in DMEM with 10% FBS (without antibiotic), and incubated with RAW 264.7 macrophages (1.5 ml/well) for 0.5 h. In total, 100 μg/ml gentamicin (for MSSA ATCC25923) or 50 μg/ml lysostaphin (for MRSA ATCC43300 and virulent CVCC546) was added to the cells and incubated for 1 h to remove non-phagocytosed bacteria. After washing twice with PBS, the RAW 264.7 macrophages were treated with different extracellular concentrations of MP1102, NZ2114, and vancomycin (0.001–100 or 1000 × MIC) for 24 h, washed again and lysed with Hanks buffered saline solution (0.1% bovine serum albumin and 0.1% Triton-X). The numbers of intracellular bacteria were measured at 0 h and 24 h using the above method.

### Determination of cytokine production using an ELISA

To investigate the effects of MP1102 and NZ2114 on cytokine levels in cells infected with *S. aureus*, RAW264.7 cells (2.5 × 10^5^ cells/ml) seeded into 12-well plates were incubated with MRSA ATCC43300 (MOI = 100:1) for 30 min followed by treatment with 50 μg/ml lysostaphin for 1 h to remove non-phagocytosed bacteria. The cells were then treated with 25 μg/ml MP1102, NZ2114 and vancomycin for 12 h and 24 h. Cell-free culture supernatants were collected and the concentrations of cytokines (TNF-α, IL-1β and IL-10) were determined by Jiaxuan Biotech. Co. Ltd. (Beijing, China) using an ELISA kit^58,59^.

### *In vivo* efficacy of MP1102 and NZ2114 in the mouse peritonitis model

Mice were bred in appropriate conventional animal care facilities and experiments were performed in accordance with the Animal Care and Use Committee of the Feed Research Institute of Chinese Academy of Agricultural Sciences (CAAS). The protocols were approved by the Laboratory Animal Ethical Committee and the Inspection of the Feed Research Institute of CAAS (AEC-CAAS-20090609).

The mice were infected by peritoneal injection with different *S. aureus* strains, including MSSA ATCC25923 (5 × 10^8^ CFU), MRSA ATCC43300 (5 × 10^8^ CFU) and high virulent CVCC546 (5 × 10^7^ CFU), and treated with a single dose of drugs (5 mg/kg for ATCC25923, 20, 30 and 40 mg/kg for ATCC43300 and CVCC546) at 10 min after infection. Twenty-four hours after treatment, the mice were killed and peritoneal fluids were obtained by washing with 5 ml ice-cold PBS. The total number of bacteria in the fluids was determined before any further procedures and then divided into two equal fractions of 1.5 ml each for the following purposes: i) for extracellular bacteria quantification, one fraction was centrifuged for 10 min at 4 °C (300 g) and the supernatant CFU count was quantified and ii) for intracellular bacteria quantification, the other fraction was centrifuged for 5 min at 4 °C (1,000 rpm). The cells were collected and treated with 100 μg/ml gentamicin (MSSA ATCC25923) or 50 μg/ml lysostaphin (MRSA ATCC43300 and high virulent CVCC546) for 1 h at 37 °C to kill extracellular bacteria. The cells were washed twice with ice-cold PBS to remove any anti-extracellular bacterial drug. Subsequently, the cells were lysed and counted as described above for the *in vitro* experiment method^14,54^.

### Statistical analyses

The Hill equation was used to analyze the dose-effect relationships of the intracellular and extracellular activities of MP1102 and NZ2114 against *S. aureus*. The E_max_, C_s_ and goodness of fit (R^2^) were calculated by non-linear regression using GraphPad Prism version 6 (GraphPad Prism Software, San Diego, CA, USA). The other statistical procedures, means and SEMs were performed and calculated using IBM SPSS Statistics 21.0.

## Electronic supplementary material

Supplementary Information
