# Supplementary material for: Increased intracellular activity of MP1102 and NZ2114 against Staphylococcus aureus in vitro and in vivo
Source: Sci Rep. 2018 Mar 9;8:4204. doi: 10.1038/s41598-018-22245-5 (PMC5844868; doi:10.1038/s41598-018-22245-5)
Supplement: Supplementary file 1 — Supplementary Information [file 41598_2018_22245_MOESM1_ESM.pdf]

Increased intracellular action of MP1102 and NZ2114 against *Staphylococcus aureus in vitro* and *in vivo*

Xiao Wang[1,2*], Xiumin Wang[1,2], Da Teng[1,2*], Ruoyu Mao[1,2], Ya Hao[1,2], Na Yang[1,2], Zhanzhan Li[1,2], Jianhua Wang[1,2**]**

[1] Key Laboratory of Feed Biotechnology, Ministry of Agriculture, Beijing 100081, P. R. China. [2] Gene Engineering Laboratory, Feed Research Institute, Chinese Academy of Agricultural Sciences, Beijing 100081, P. R. China. * Equal contributors into this paper. ** Correspondence and requests for materials should be addressed to X.M.W. (email: wangxiumin@caas.cn) and J.H.W. (email: wangjianhua@caas.cn)

SUPPORTING INFORMATION

Supplementary 1: Materials and Methods

Antimicrobial susceptibility testing of *S. aureus*. Antimicrobial susceptibility testing of *S. aureus* (ATCC43300 and CVCC546) was performed by Kirby Bauer disc diffusion method according to the standards procedures recommended by Clinical Laboratory Standards Institute (CLSI 2012)[1,2]. The used 22 antibiotics included: 1) aminoglycosides (amikacin, neomycin, gentamicin, kanamycin, and streptomycin), 2) quinolones (ciprofloxacin, norfloxacin), 3) sulfonamides (sulfisoxazole), 4) tetracyclines (tetracycline), 5) polypeptides (bacitracin and polymyxin B), 6) penicillins (penicillin, amoxicillin, ampicillin, oxacillin), 7) lincosamides (lincomycin), 8) macrolides (erythromycin and azithromycin), 9) amphenicols (chloramphenicol), 10) glycopeptides (vancomycin), and 11) cephalosporins (cefazolin and cefotaxime) (Qianshun Bio-tech, Beijing).

SCC*mec* and *spa* typing of *S. aureus*. For rapid DNA extraction, one to five bacterial colonies were

suspended in 50 μl of sterile distilled water and heated at 100°C for 10 min. Then the solution was frozen in -80°C for 30 min and heated at 100°C for 10 min. After centrifugation at 5000 rpm/min for 2 min. A total of 2 μl of the supernatant was used as a template in a 50-μl PCR. The SCC*mec* and x region of the *spa* gene was amplified by PCR with primers in Supplementary Table 1[3-5]. The x region of the spa genes was sequenced by Sangon Biotech (Shanghai, China) and the types were determined with the database accessible via http://spa.ridom.de/spatypes.shtml.

Growth curves of *S. aureus*. The *S. aureus* ATCC25923, ATCC43300 and CVCC546 strains were used to evaluate growth rate in Mueller – Hinton broth (MHB)[6]. Briefly, overnight cultures in MHB was inoculated into 50 ml of fresh MHB (with the inoculum size of 1% (v/v))[7]. All flasks were incubated at 37°C for 15 h with shaking (250 rpm). Aliquots (2 ml) were taken out per hour to determine the culture turbidity at 600 nm in a TU-1810 ultraviolet visible spectrophotometer. It was performed for three times.

FITC-labeled peptides. The FITC-labeled peptides were carried out in ChinaPeptides Co., Ltd. (Shanghai, China) as previously described[8]. The peptides (MP1102 or NZ2114) were dissolved in 0.2 M PBS (pH 8.4) containing 1.1 equivalent of FITC. The mixture was stirred at room temperature for 12 h to complete the reaction. The reaction product was then dialyzed in dialysis tubing MD18 (with a molecular weight cut-off 2.0 kDa) at 4°C for 48 h with an exchange of ddH$_2$O per 12 h to remove free FITC. The fluorescence intensity of FITC in dialyzed ddH$_2$O was monitored to assure the complete removal of free FITC. Finally, the product was lyophilized in a freezer dryer for 36 h. The procedure was kept away from light.

References:

[1] Kahsay, A., Mihret, A., Abebe, T., Andualem, T. Isolation and antimicrobial susceptibility pattern of *Staphylococcus aureus* in patients with surgical site infection at Debre Markos Referral Hospital, Amhara

Region, Ethiopia. *Arch Public Health* **72**, *16* (2014).

[2] Wikler, M. A., *et al.* Performance standards for antimicrobial sensitivity testing: seventeenth informational supplement. CLSI. **26**, 1–177 (2007).

[3] Zhang, K., McClure, J. A., Elsayed, S., Louie, T., Conly, J. M. Novel multiplex PCR assay for characterization and concomitant subtyping of staphylococcal cassette chromosome *mec* types I to V in methicillin-resistant *Staphylococcus aureus*. *J Clin Microbiol* **43**, 5026–5033 (2005).

[4] Rong, D., Wu, Q., Xu, M., Zhang, J., Yu, S. Prevalence, virulence genes, antimicrobial susceptibility, and genetic diversity of *Staphylococcus aureus* from retail aquatic products in China. *Front Microbiol* **8**, 714 (2017).

[5] Strommenger, B., *et al. spa* Typing of *Staphylococcus aureus* as a frontline tool in epidemiological typing. *J Clin Microbiol* **46**, 574-81 (2008).

[6] Diarra, M. S., Petitclerc, D., Lacasse, P. Response of *Staphylococcus aureus* isolates from bovine mastitis to exogenous iron sources. *J Dairy Sci* **85**, 2141–2148 (2002).

[7] Buchanan, R. L., *et al.* Response surface models for the effects of temperature, pH, sodium chloride, and sodium nitrite on the aerobic and anaerobic growth of *Staphylococcus aureus* 196e1. *J Food Safety* **13**:159–175 (1993).

[8] Sun, X. X., *et al.* Fluorescence characterization of the thermal stability of collagen mimic peptides. *Chinese Chem Lett* **28**, 963–967 (2017).

Supplementary 2: Tables

Table 1 Primers used in SCC*mec* and *spa* typing of *S. aureus*

**Name**	**Primer (5'→3')**	**Amplicon size (bp)**	**Reference**
**SCC*mec* type**			
**SCCmecI-F**	GCTTTAAAGAGTGTCGTTACAGG	613	3, 4
**SCCmecⅠ-R**	GTTCTCTCATAGTATGACGTCC		
**SCCmecⅡ-F**	CGTTGAAGATGATGAAGCG	398	
**SCCmecⅡ-R**	CGAAATCAATGGTTAATGGACC		
**SCCmecⅢ-F**	CCATATTGTGTACGATGCG	280	
**SCCmecⅢ-R**	CCTTAGTTGTCGTAACAGATCG		
**SCCmecⅣa-F**	GCCTTATTCGAAGAAACCG	776	
**SCCmecⅣa-R**	CTACTCTTCTGAAAAGCGTCG		
**SCCmecⅣb-F**	TCTGGAATTACTTCAGCTGC	493	
**SCCmecⅣb-R**	AAACAATATTGCTCTCCCTC		
**SCCmecⅣc-F**	ACAATATTTGTATTATCGGAGAGC	200	
**SCCmecⅣc-R**	TTGGTATGAGGTATTGCTGG		
**SCCmecⅣd-F**	CTCAAAATACGGACCCCAATACA	881	
**SCCmecⅣd-R**	TGCTCCAGTAATTGCTAAAG		
**SCCmecⅤ-F**	GAACATTGTTACTTAAATGAGCG	325	
**SCCmecⅤ-R**	TGAAAGTTGTACCCTTGACACC		
***mecA* gene**			
**mecA-F**	GTGAAGATATACCAAGTGATT	147	3
**mecA-R**	ATGCGCTATAGATTGAAAGGAT		
**mecI-F**	CCCTTTTTATACAATCTCGTT	146	
**mecI-R**	ATATCATCTGCAGAATGGG		
***Spa* type**			
**Spa-x-F**	TAAAGACGATCCTTCGGTGAGC	-	5
**Spa-x-R**	CAGCAGTAGTGCCGTTTGCTT		

-: no data. The primers were synthesized by Sangon Biotech (Shanghai, China).

Table 2 Antimicrobial susceptibility patterns of *S. aureus* ATCC43300 and CVCC546

**Antibiotics**	***S. aureus* ATCC43300**	***S. aureus* CVCC546**
Vancomycin	S	S
Lincomycin	R	I
Amoxicillin	R	S
Ciprofloxacin	S	S
Amikacin	R	S
Ampicillin	R	S
Oxacillin	R	S
Erythromycin	R	I
Tetracycline	I	R
Bacitracin	I	R
Norfloxacin	S	S
Polymyxin B	R	R
Sulfisoxazole	R	R
Neomycin	R	I
Azithromycin	R	S
Kanamycin	R	I
Streptomycin	I	S
Cefotaxime	I	I
Gentamicin	R	S
Chloramphenicol	S	S
Cefazolin	S	S
Penicillin	R	I

R: resistant; I: Intermediate; S: susceptible.

Table 3 Sources, types and susceptibilities of different *S. aureus* strains

S. aureus	**Source**	**SCC*mec* type**	***Spa* type**	**Antibiotic resistance profile**	**Biosafety level**
Methicillin-susceptible ATCC25923	Clinical isolate	-	-	-	II
Methicillin-resistant ATCC43300	Clinical isolate	II	t007	Methicillin, oxacillin[a]	II
Highly virulent CVCC546	Isolation from a pig in Bejing, China	-	t034	Tetracycline, bacitracin, polymyxin B and sulfisoxazole[b]	III

-: negative. a: the ATCC website. b: in this study.

Table 4 Effect of cathepsin B on the MICs of MP1102, NZ2114 against *S. aureus* resistant ATCC43300

	**MIC (μg/ml)**	
	Buffer + AMP	Buffer + cathepsin B + AMP
MP1102	0.25	0.25
NZ2114	0.5	0.5

Supplementary 3: Figures

A

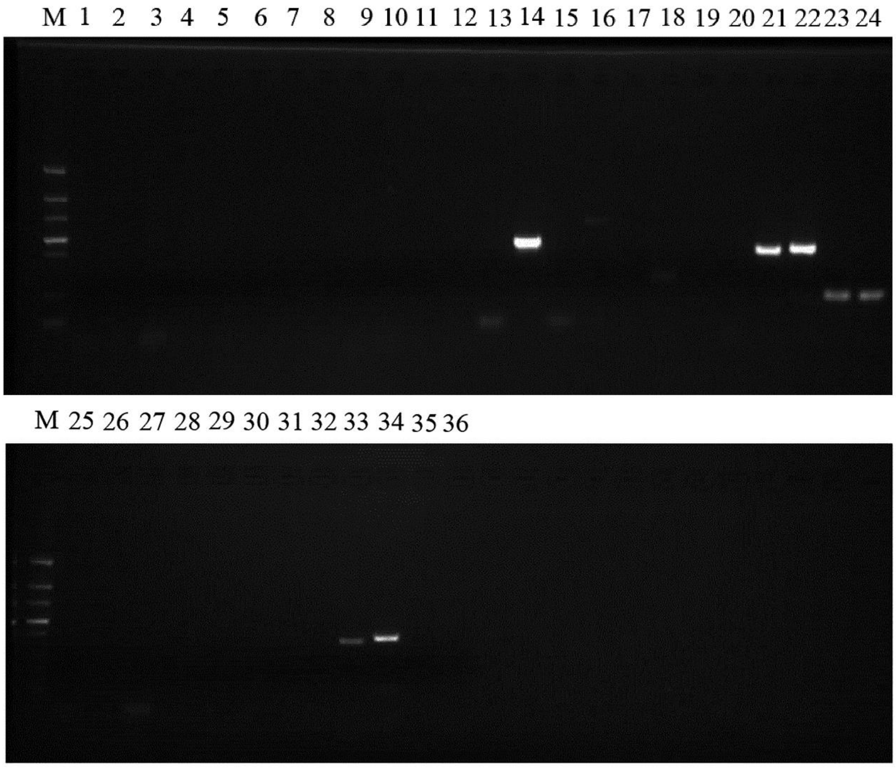

B

S. aureus ATCC43300

TAATTAAAGACGATCCTTCGGTGAGCAAAGAAATTTTAGCAGAAGCTAAAAAGCTAAA
CGATGCTCAAGCACCAAAAGAGGAAGACAACAACAAGCCTGGCAAAGAAGACAACA
ACAAGCCTGGTAAAGAAGACGGCAACAAACCTGGTAAAGAAGACGGCAACAAACCT
GGTAAAGAAGACGGCAACAAACCTGGTAAAGAAGACGGCAACAAACCTGGTAAAGA
AGACAACAAAAAACCTGGCAAAGAAGATGGCAACAAACCTGGTAAAGAAGACGGCA
ACAAGCCTGGTAAAGAAGACGGCAACGGAGTACATGTCGTTAAACCTGGTGATACAGT
AAATGACATTGCAAAAGCAAACGGCACTACTGCTG
t007,15-12-16-16-16-16-02-25-17

S. aureus CVCC546

TAAAGACGATCCTTCGGTGAGCAAAGAAATTTTAGCAGAAGCTAAAAAGCTAAACGAT
GCTCAAGCACCAAAAGAGGAAGACAACAACAAGCCTGGTAAAGAAGACGGCAACAA
ACCTGGTAAAGAAGACAACAAAAAACCTGGCAAAGAAGATGGCAACAAACCTGGTA
AAGAAGACAACAAAAAACCTGGCAAAGAAGATGGCAACAAACCTGGTAAAGAAGAC
AACAAAAAACCTGGTAAAGAAGATGGCAACAAGCCTGGTAAAGAAGATGGCAACAA
ACCTGGTAAAGAAGACGGCAACGGAATACATGTCGTTAAACCTGGTGATACAGTAAAT
GACATTGCAAAAGCAAACGGCACTACTGCTG
t034,08-16-02-25-02-25-34-24-25

Fig. S1

Figure 1 PCR assay identifies SCC*mec* and *spa* types and subtypes and simultaneously detects the

methicillin resistance (*mecA* gene). **(A)** Gel electrophoresis. M: DNA Marker II; 1-12: *S. aureus* ATCC25923; 13-24: *S. aureus* ATCC43300; 25-36: *S. aureus* CVCC546. 1, 13, 25: SCC*mec* I; 2, 14, 26: SCC*mec* Ⅱ; 3, 15, 27: SCC*mec* Ⅲ; 4, 16, 28: SCC*mec* Ⅳe; 5, 17, 29: SCC*mec* Ⅳe; 6, 18, 30: SCC*mec* Ⅳc; 7, 19, 31: SCC*mec* Ⅳe; 8, 20, 32: SCC*mec* Ⅴ; 9, 21, 33: spa-x-1; 10, 22, 34: spa-x-2; 11, 23, 35: *mecA*; 12, 24, 36: *mec* I. **(B)** The x region of the *spa* gene sequence.

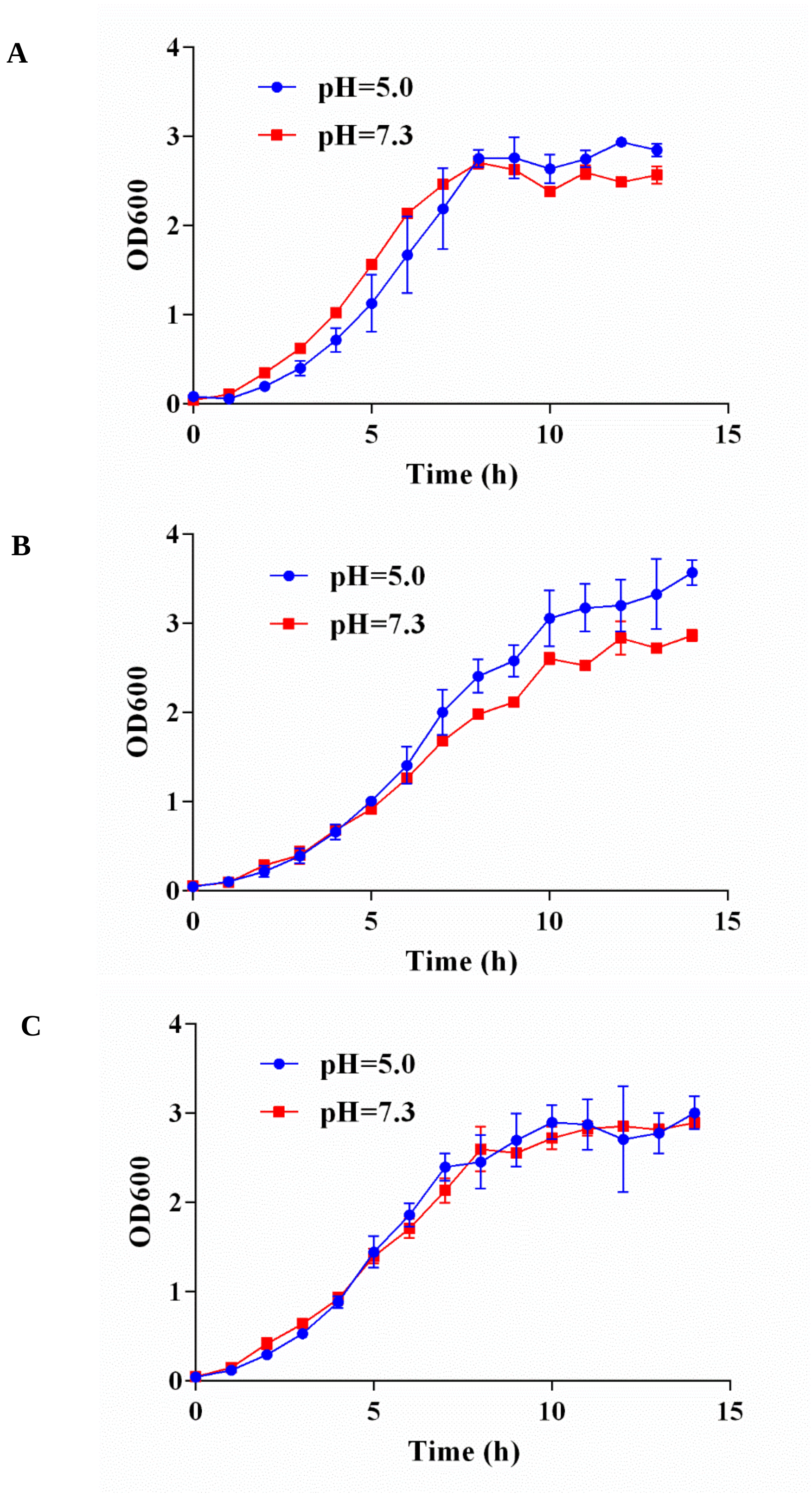

Fig. S2

Figure 2 Growth curves of *S. aureus* ATCC25923 **(A)**, ATCC43300 **(B)** and CVCC546 **(C)** at pH 5.0 and pH 7.3.

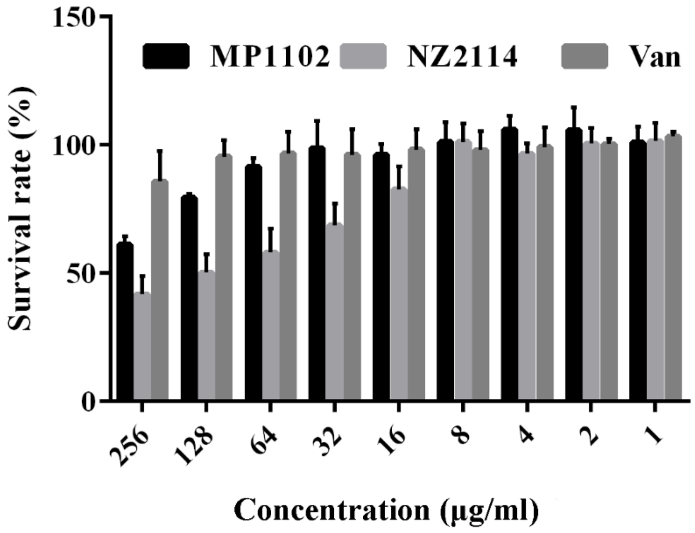

Fig. S3

Figure 3 Viability of RAW 264.7 cells incubated with NZ2114 and MP1102 for 24 h. RAW 264.7 cells were incubated with 1-256 µg/ml for 24 h and subsequently cell viability was determined using the MTT assay. Results are means ± S.D. for three experiments.

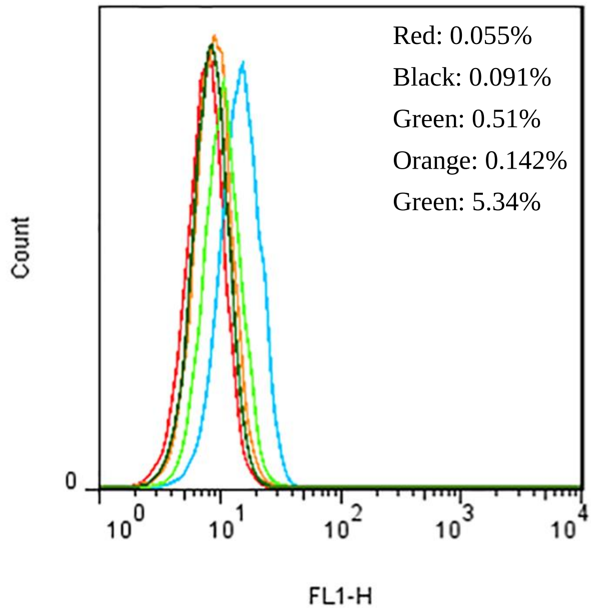

Fig. S4

Figure 4 Effect of MP1102 and NZ2114 on the cell membrane of RAW 264.7 cells. Cells were incubated with different concentrations of MP1102 and NZ2114 for 24 h prior to staining with 5 µg/ml PI at 37°C for 10 min. PI fluorescence in RAW 264.7 cells was measured by flow cytometry. Red line: control; black line: 25 µg/ml MP1102; green line: 250 µg/ml MP1102; orange line: 25 µg/ml NZ2114; green line: 250 µg/ml NZ2114.
